# Supplementary figures and images for: A Decade-Long Evaluation of Neonatal Septicaemic Escherichia coli: Clonal Lineages, Genomes, and New Delhi Metallo-Beta-Lactamase Variants
Source: Microbiol Spectr. 2023 Jun 27;11(4):e05215-22. doi: 10.1128/spectrum.05215-22 (PMC10434172; doi:10.1128/spectrum.05215-22)

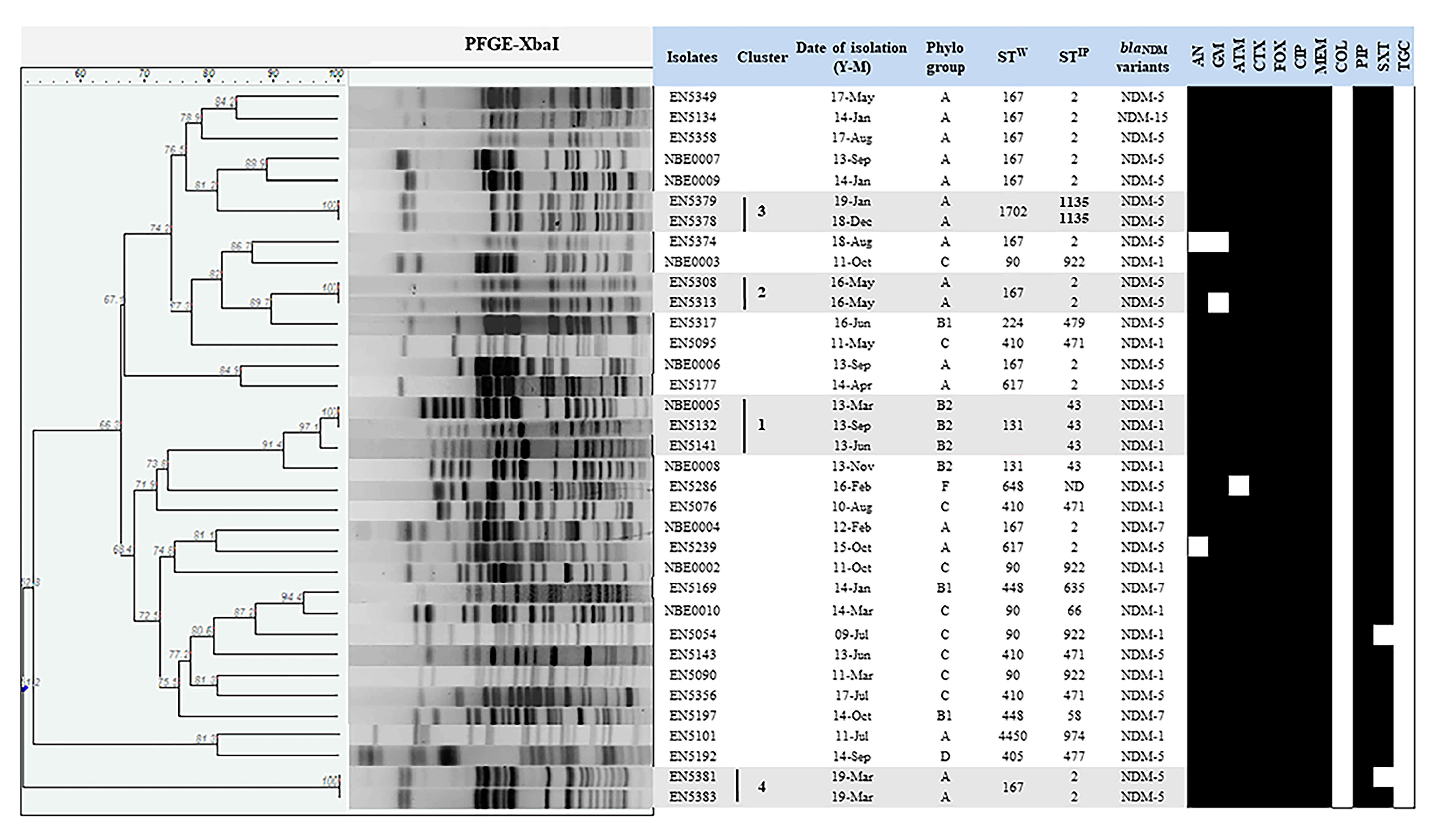

Supplement: Supplemental file 1 — Fig. S1. Download spectrum.05215-22-s0001.tif, TIF file, 9.2 MB [file spectrum.05215-22-s0001.tif]

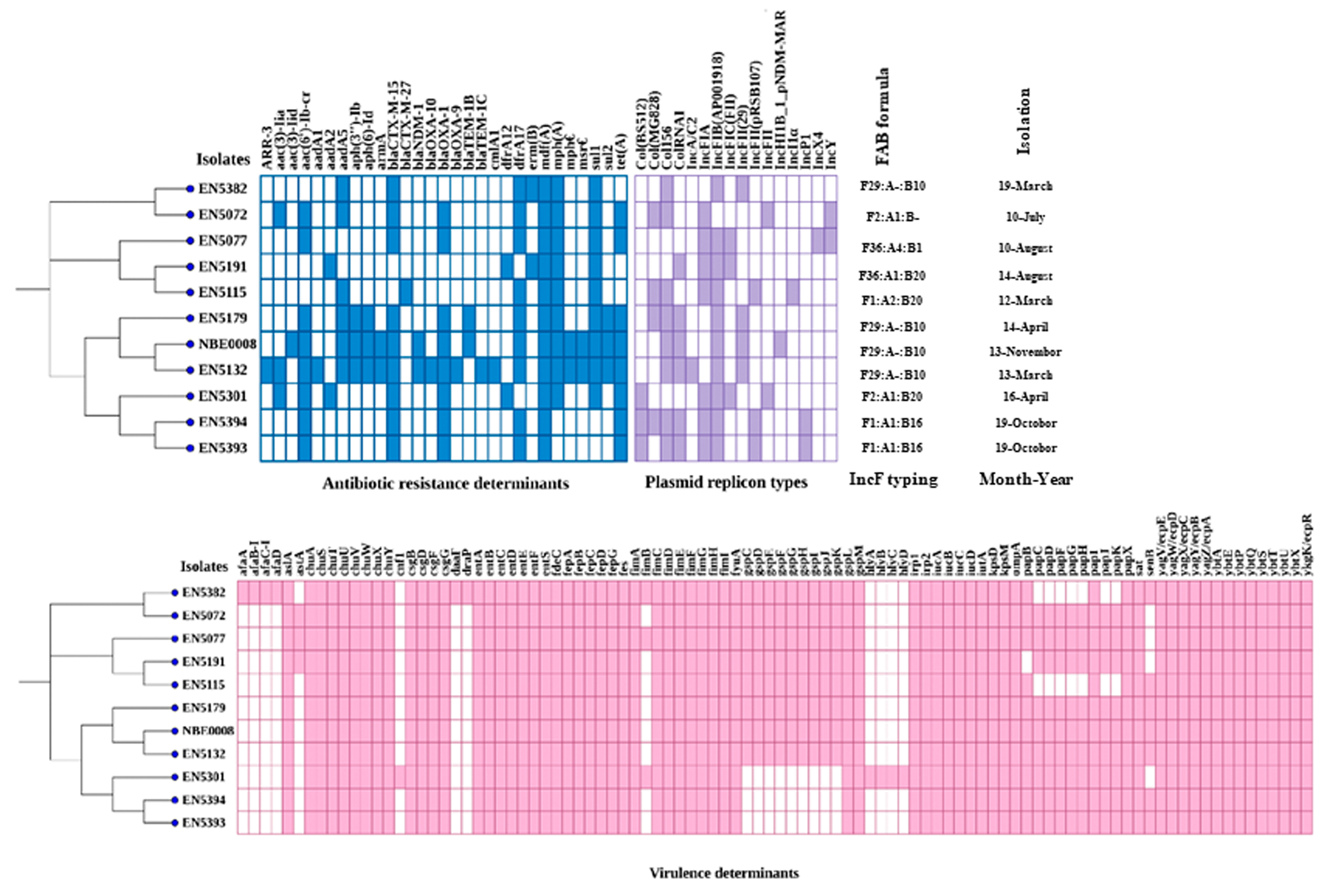

Supplement: Supplemental file 2 — Fig. S2. Download spectrum.05215-22-s0002.tif, TIF file, 5.8 MB [file spectrum.05215-22-s0002.tif]
