# Supplementary material for: A Decade-Long Evaluation of Neonatal Septicaemic Escherichia coli: Clonal Lineages, Genomes, and New Delhi Metallo-Beta-Lactamase Variants
Source: Microbiol Spectr. 2023 Jun 27;11(4):e05215-22. doi: 10.1128/spectrum.05215-22 (PMC10434172; doi:10.1128/spectrum.05215-22)
Supplement: Supplemental file 3 — Supplemental text, legends of Fig. S1 and S2, and Tables S1 to S3. Download spectrum.05215-22-s0003.docx, DOCX file, 0.04 MB [file spectrum.05215-22-s0003.docx]

# Supplementary Material

# Title: A Decade-long Evaluation of Neonatal Septicaemic *Escherichia coli*: Clonal Lineages, Genomes and New Delhi Metallo-β-Lactamase Variants

Amrita Bhattacharjee^a^, Kirsty Sands^b,c^, Shravani Mitra^a^, Ritojeet Basu^d^, Bijan Saha^e^, Olivier Clermont^f,g^, Shanta Dutta^a^, Sulagna Basu^a*^

**Affiliations:**

*^a^Division of Bacteriology, ICMR-National Institute of Cholera and Enteric Diseases, P33 CIT Road, Scheme XM, Beliaghta, Kolkata, India;*

*^b^Division of Medical Microbiology, Institute of Infection and Immunity, Cardiff University, UK*

*^c^Ineos Oxford Institute of Antimicrobial Research, Department of Biology, University of Oxford, UK*

*^d^Department of Economics, University of Warwick, Gibbet Hill Road, Coventry CV4 7AL, UK*

*^e^Department of Neonatology, Institute of Post-Graduate Medical Education & Research and SSKM Hospital, Kolkata 700020, India.*

*^f^Université de Paris, IAME, UMR1137, INSERM, Paris, France.*

*^g^Université Sorbonne Paris Nord, IAME, Paris, France.*

*Corresponding author:

Sulagna Basu, Division of Bacteriology, ICMR-National Institute of Cholera and Enteric Diseases, P-33, C.I.T. Road, Scheme XM, Beliaghata, Kolkata-700010, West Bengal, India.

Telephone: +91-33-23537469/7470, 23705533/4478/0448; ext: 3055. Fax: +91-33-2363, 2370 5066

Running Head: Epidemic Clones ST167,131 of *E. coli* Causing Sepsis

**Supplementary Text**

**Method for whole genome sequence (WGS) analysis**

Genomic DNA was extracted from isolates for short-read sequencing using Wizard® Genomic DNA Purification Kit, Promega. The concentration, along with purification (1.8-2 at OD_260/280_) of the DNA was checked in a Nanodrop spectrometer (Eppendorf µCuvetter® G1.0) followed by Invitrogen Qubit 4 fluorometer (Thermo Fisher Scientific). DNA library preparation was carried out for paired-end sequencing (2× 150 cycles) using Nextera XT kit (Illumina Inc., San Diego, CA). Whole genome sequencing was executed on Illumina platform (Illumina Inc., San Diego, CA) by Illumina NovaSeq 6000 sequencer. Fastq sequence reads were assembled into contigs using shovill (v0.9.0) following QC and base trimming using fastqc (v0.11.8) and trimgalore (v0.5.0), respectively.

Using the short read data, the following online-based services were achieved, i.e., i) Phylogroup by Clermont typing (<http://clermontyping.iame-research.center/>), ii) MLST for sequence type identification (<https://cge.food.dtu.dk/services/MLST/>), iii) SeroTypeFinder (<https://cge.food.dtu.dk/services/SerotypeFinder/>) for serotype, iv) VirulenceFinder and VFDB for virulence genes (<https://cge.food.dtu.dk/services/VirulenceFinder/>) v) FimTyper for fimH allele determination (<https://cge.food.dtu.dk/services/FimTyper/>), vi) CHTyper (<https://cge.food.dtu.dk/services/CHTyper/>) for C-H type vii) ResFinder (<https://cge.food.dtu.dk/services/ResFinder/>) and Resistant Gene Identifier (<https://card.mcmaster.ca/analyze/rgi>) for resistant genes, viii) PlasmidFinder for detection of plasmid replicon types (<https://cge.food.dtu.dk/services/PlasmidFinder/>) ix) the integral site for nomenclature of the integron sequences ((<http://integrall.bio.ua.pt>), x) ISfinder for IS elements (<https://isfinder.biotoul.fr/>). All the PCR data were corroborated with the genome data where it was applicable.

Two phylogenies were performed based on single nucleotide polymorphism (SNP), one for all study ST^W^131 and the other with ST^W^167 isolates (*bla*_NDM_**^+ve^**) in addition to both ST^W^131 and ST^W^167 NCBI genomes selected following a literature search in PubMed. Snippy (v4.6.0) with the --ctgs parameter was used to map the short reads against an internal reference, snippy core was used to generate a SNP alignment and snippy-clean was performed to standardise the alignment. Gubbins (v2.3.4) was used to remove recombination events and snp-sites (v2.5.1) were used to extract the WG SNP sites. IQtree (v2.0) was used to generate a SNP phylogeny and iTOL (v6.0) was used to annotate and visualise the phylogenetic tree. Pairsnps (v0.0.7) was used to generate a SNP matrix and exported to .csv. A core genome phylogenetic tree was generated for all study *bla*_NDM_**^+ve^** isolates using Panaroo (v.1.2.8) to create a core genome alignment and IQtree (v2.0) was used to generate a maximum likelihood tree with 1000 bootstraps.

**Supplementary Figure legends**

**Supplementary Figure S1.** Genetic relatedness of the *bla*_NDM_**^+ve^** *E. coli* isolates causing sepsis in neonates over ten years. Digestion of genomic DNA by Xba1 enzyme created a distinct pattern for each isolate. *Salmonella* serotype Braenderup H9812 was used as the reference standard. The analysis followed Dice's similarity coefficient and UPGMA (the position tolerance and optimisation were set at 1.5 and 1.5%, respectively).

ST^W,^ sequence type in Warwick scheme; ST^IP^, sequence type in Institut Pasteur scheme; Y-M, year and month of sample isolation; AN, amikacin; GN, gentamicin; ATM, aztreonam; CTX, cefotaxime, FOX, cefoxitin; CIP, ciprofloxacin; MEM, meropenem; COL, colistin; PIP, piperacillin; SXT, sulfamethoxazole-trimethoprim; TGC, tigecycline. Black coloured box indicates non-susceptibility to respective antibiotics.

**Supplementary Figure S2.** Core-genome phylogeny of ST^W^131 isolates found in this study

**Supplementary Table S1**. Different sequence types in Institut Pasteur scheme with their clonal complex (STc) in institut Pasteur (ST^IP^) and Warwick (ST^W^) schemes linked to their corresponding phylogroups.

| **Phylogroup** | **ST in Institut Pasteur (ST^IP^)** | **STc in Institut Pasteur**  **(ST^IP^c)** | **STc in Warwick**  **(ST^W^c)** | **Association with NDM** |
| --- | --- | --- | --- | --- |
| **A** | ST^IP^2  ST^IP^974 | ST^IP^c2  ST^IP^c974 | **ST^W^c10**  ST^W^c4450 | NDM-5, NDM-7, NDM-15  NDM-1 |
| **B2** | ST^IP^43 | ST^IP^c43 | **ST^W^c131 (Sub I)** | NDM-1 |
|  | ST^IP^506 | ST^IP^c43 | **ST^W^c131** |  |
|  | ST^IP^4 | ST^IP^c4 | ST**^W^**c73 (Sub II) |  |
|  | ST^IP^6 | ST^IP^c6 | ST**^W^**c14(Sub VII) |  |
|  | ST^IP^33 | ST^IP^c33 | ST**^W^**c127 (Sub III) |  |
|  | ST^IP^36 | ST^IP^c36 | ST**^W^**c12 (Sub VI) |  |
|  | ST^IP^53 | ST^IP^c6 | ST**^W^**c14 |  |
|  | ST^IP^129 | ST^IP^c26 | ST**^W^**c141(Sub IV) |  |
|  | ST^IP^686 | ST^IP^c36 | ST**^W^**c12 |  |
| **B1** | ST^IP^58 | ST^IP^c58 | ST**^W^**c94 | NDM-7 |
|  | ST^IP^635 | ST^IP^c58 | ST**^W^**c94 | NDM-7 |
|  | ST^IP^21 | ST^IP^c21 | ST**^W^**c155 |  |
|  | ST^IP^479 | ST^IP^c845 | ST**^W^**c224 | NDM-5 |
| **C** | ST^IP^471 | ST^IP^c7 | **ST^W^c410** | NDM-1 |
|  | ST^IP^922 | ST^IP^c66 | ST**^W^**c88 | NDM-1 |
|  | ST^IP^66 | ST^IP^c66 | ST**^W^**c88 |  |
| **D** | ST^IP^3 | ST^IP^c3 | ST**^W^**c69 |  |
|  | ST^IP^8 | ST^IP^c8 | ST**^W^**c69 |  |
|  | ST^IP^477 | ST^IP^c477 | **ST^W^c405** | NDM-5 |
|  | ST^IP^678 | ST^IP^c678 | ST**^W^**c349 |  |
| **F** | ND | ND | **ST^W^c648** | NDM-5 |

Sub, B2 Subgroup; ND, not determinable

High-risk clones were presented in bold text.

**Supplementary Table S2A.** List of published literature from different region of India focusing on neonatal sepsis

| **Journal Title** | **Year of Publication** | **Study timeline** | **State** | ***E coli* Prevalence** | **Sensitivity** | **MDR** | **Mortality** |
| --- | --- | --- | --- | --- | --- | --- | --- |
| Aetiology and Antimicrobial Resistance of Neonatal Sepsis at a Tertiary Care Centre in Eastern India: A 3 Year Study (DOI: [10.1007/s12098-010-0272-1](https://doi.org/10.1007/s12098-010-0272-1)) | 2011 | 2007 to 2009 | Kolkata, West Bengal | 15 | Ampicillin, Gentamicin,  Amikacin and Cefotaxime | NA | NA |
| Multi-drug resistant gram negative bacilli causing early neonatal sepsis in India (DOI: [10.1136/archdischild-2011-300097](https://doi.org/10.1136/archdischild-2011-300097)) | 2012 | January 2008 to December 2009 | Kolkata, West Bengal | 24 | Cefotaxime and Gentamicin (Partially) | Present | 21% ( including other Gram-negative organisms) |
| Bacteriological Profile of Neonatal Sepsis in a Tertiary-care Hospital of Northern India (PMID: 25691192) | 2015 | January 2008  to December 2012 | Northern India | 16% (Total=168) | Ampicillin, Piperacillin, Imipenem, Cefotaxime,  Ciprofloxacin, Amikacin, Gentamicin | NA | NA |
| Bacteriological Profile of Neonatal Septicemia in a Tertiary Care Hospital from Western India (DOI: [10.4103/0974-777X.154444](https://doi.org/10.4103%2F0974-777X.154444)) | 2015 | NA | Pune, Maharashtra | 16.7%  (Total=48) | Imipenem, Amikacin, Ampicillin, Cefotaxime,  Ciprofloxacin, Cotrimoxazole, Gentamicin (Partially) | NA | NA |
| Characterisation and antimicrobial resistance of sepsis pathogens in neonates born in tertiary care centres in Delhi, India: a cohort study  (DOI:[10.1016/S2214-109X(16)30148-6](http://dx.doi.org/10.1016/S2214-109X(16)30148-6)) | 2016 | July 2011 to Feb 2014 | Delhi | 14%  (Total=1005) | Cephalosporins (47%)  Carbapenems (15%)  MDR (38%) | 38% | 61% (for *E. coli*) |
| - Evaluation of co-transfer of plasmid-mediated fluoroquinolone resistance genes and *bla*_NDM_ gene in *Enterobacteriaceae* causing neonatal septicaemia (DOI:org/10.1186/s13756-019-0477-7) | 2019 | January 2012 to June2014 | Kolkata, West Bengal | 22% (Total=73) | Colistin, Tigecycline | 97% | NA |
| Changing Trends of Antimicrobial Resistance in Neonatal Sepsis: Experience from a Tertiary Care Hospital from West Bengal, India  (DOI:[10.7860/JCDR/2021/49015.15136](http://dx.doi.org/10.7860/JCDR/2021/49015.15136)) | 2021 | March 2017 to February 2019 | West Bengal, | 6.4% (Total=156) | Meropenem, Ciprofloxacin, Colistin, Tigecycline | NA | NA |
| Bacterial Etiology of Neonatal Sepsis, Antibiotic Susceptibility Profile, and Associated Factors at Burdwan Medical College, Burdwan, West Bengal, India (DOI:10.1055/s-0041-1731305) | 2021 | March 2017 to  February 2018. | West Bengal | 22.8%  (Total=118) | Meropenem, Colistin, Levofloxacin, Amikacin, Cotrimoxazole | NA | NA |
| - Bacteriological Profile and Outcome of Culture-Positive Neonatal Sepsis in a Special Newborn Care Unit Setting, Odisha ( DOI: [10.7759/cureus.25539](https://doi.org/10.7759/cureus.25539)) | 2022 | May 2017 to October 2019 | Odisha | 19.1% (Total=115) | Colistin | 35% | 7% |

NA, Not available; MDR, multidrug-resistance

**Supplementary** **Table S2B.** List of published literature from different countries focusing on neonatal sepsis

| **Journal Title** | **Year of Publication** | **Study timeline** | **State/ Country** | **Isolate number** | **Sensitivity** | **Phylogroup/Sequence type** | **Resistome** | **Virulome** | **Plasmid Types** |
| --- | --- | --- | --- | --- | --- | --- | --- | --- | --- |
| - Virulence factors of *Escherichia coli* isolated from female reproductive tract infections and neonatal sepsis (DOI: [10.1155/S1064744901000333](https://doi.org/10.1155/s1064744901000333)) | 2001 | NA | Texas | 45 | NA | NA | NA | *pap, pil, foc, sfa, uca, dra* | NA |
| Comparative Study of Virulence Traits of *Escherichia coli* Clinical Isolates Causing Early and Late Neonatal Sepsis (DOI: [10.1128/JCM.01682-07](https://doi.org/10.1128%2FJCM.01682-07)) | 2007 | 1987 to 2006 | Barcelona | 47 | NA | B2(51%), D(32%), A(11%), B1(6%) | NA | *fimA* (91%), *ibeA* (68%), *iucC* (60%), *papEF* (57%), and *papC* (47%), *cnf1* and *sat1* (both 2%) | NA |
| - Antimicrobial Resistance of *Escherichia coli* Strains Causing Neonatal Sepsis between 1998 and 2008 ( DOI: [10.1159/000337062](https://doi.org/10.1159/000337062)) | 2012 | 1995 to 2008 | Barcelona | 61 | NA | NA | *bla*_CTX-M-15_,  *bla*_CTX-M-14_, *bla*_TEM-Like,_  *_aac(3)-II,_ tetA, tetB, tetC,tetG, dfrA1, dfrA17, dfrA12, sulII* | NA | NA |
| - Characterization of CTX-M-14 and CTX-M-15 Producing Escherichia coli Strains Causing Neonatal Sepsis - ( DOI: [10.1089/mdr.2013.0190](https://doi.org/10.1089/mdr.2013.0190)) | 2014 | 2008 | Spain | 2 | Resistant to Cephalosporin, Ciprofloxacin, Tetracycline, Chloramphenicol, Trimethroprim-sulfamethoxazole | B2/ST705  D/ST156 | *bla*_CTX-M-14_*, bla*_CTX-M-15_*, aac(3)-II, cml1, tetA_,_ dfrA12* and *sul2* | *hlyA, sat1, papA, papC, papEF, papGII, papGIII, prs, malX, focG, iha, fyuA, fimA, hra, iutA, iucC, sfaS.* | IncFIA |
| - Early-onset neonatal infections n Australia and New Zealand, 2002–2012 ( DOI: [10.1136/archdischild-2017-314671](https://doi.org/10.1136/archdischild-2017-314671)) | 2018 | January 2002 to December 2012 | Australia and New Zealand | 114 | NA | NA | NA | NA | NA |
| Antibiotic resistance and molecular characterization of bacteremia *Escherichia coli* isolates from newborns in the United States (DOI: [10.1371/journal.pone.0219352](https://doi.org/10.1371/journal.pone.0219352)) | 2019 | 2006 to 2016. | Oklahoma, United States | 43 | Resistant to Ampicillin, Gentamicin, Tobramycin, Trimethoprim/sulfamethoxazole | B2 (81%), D (12%)/ST95, ST131, ST1193, ST405, ST69, ST501 | *bla*_CTX-M-15_, *bla*_OXA-1_ | *fimH*, *nlpI*, *ompA* (100%)*; cnf1* (35%) *hek/hra* (35%) *hlyC* (28%)*, ibeA* (16%)*, iucC* (58%) *iroN* (44%)*, kpsMII* (88%*), papGII-III* (53%) *sfa/focDE* (21%) *and K1 capsule* (46%) | NA |
| Molecular characteristics of the new emerging global clone ST1193 among clinical isolates of Escherichia coli from neonatal invasive infections in China (DOI: [10.1007/s10096-020-04079-0](https://doi.org/10.1007/s10096-020-04079-0)) | 2020 | September 2009 to June 2015 | China | 41 | Resistant to Ciprofloxacin, Amoxicillin, Sulfonamides, and  Tetracycline. | ST1193, ST95, ST62 | *bla*_CTX-M-15,-27_ and *bla*_TEM_ | NA | NA |
| - Neonatal sepsis at Mulago national referral hospital in Uganda: Etiology, antimicrobial resistance, associated factors and case fatality risk ( DOI: [10.1371/journal.pone.0237085](https://doi.org/10.1371/journal.pone.0237085)) | 2020 | January to December 2018 | Uganda | 7 | Resistant to Ampicillin, Gentamicin, Ceftriaxone and Ceftazidime) but susceptible to Carbapenem | NA | NA | NA | NA |
| Neonatal and young infant sepsis by Group B Streptococci and Escherichia coli: a single-center retrospective analysis in Germany—GBS screening implementation gaps and reductionin antibiotic resistance (DOI: [10.1007/s00431-020-03659-8](https://doi.org/10.1007/s00431-020-03659-8)) | 2020 | 2008 and 2018 | Germany | 73 | Resistant to Ampicillin, Piperacillin-Tazobactam, Cefotaxime, Gentamicin but susceptible to Meropenem | NA | NA | NA | NA |
| Prevalence of Multi-drug resistant *Escherichia coli* isolated in neonatal intensive care units in a local hospital, Minia, Egypt (DOI: [10.21608/MJMR.2022.220836](https://doi.org/10.21608/mjmr.2022.220836)) | 2020 | August 2016 to April 2017 | Egypt | 41 | resistant to Cefotaxime (100%), Linezolid (100%), Ampicillin (97.5%), Amoxicillin-Clavulanic acid (97.5%), Aztreonam (97.5%), Cefepime (97.5%), Meropenem (97.5), Ampicillin-Sulbactam,(82.9%), Amikacin (75.6%), Gentamicin (73.1%), Piperacillin-Tazobactam (73.1%) and Ciprofloxacin (60.9%), least resistance was found to Imipenem (34.1%) and Azithromycin (34.1%) | NA | NA | *fimH, hlyA* | NA |
| - Characterization of antimicrobial-resistant Gram-negative bacteria that cause neonatal sepsis in seven low- and middle-income countries - ( DOI: [10.1038/s41564-021-00870-7](https://doi.org/10.1038/s41564-021-00870-7)) | 2021 | November 2015 to December 2017 | 7 countries in Africa and South Asia, Ethiopia, India, Nigeria, Rwanda, Pakistan, Cape town | 75 | Susceptible to Colistin and Tigecycline | ST10, ST69, ST131,  ST410 and ST517 | *bla*_CTX-M-15_ (21/75), *bla*_NDM-5_ (2/75), *bla*_OXA-181_ (1/75) | NA | ColKP3 |

NA, Not available

**Supplementary Table S3A: Two-way table of Outcome and presence of NDM**

| NDM | Mortality | | |
| --- | --- | --- | --- |
|  | 0 | 1 | Total |
| 0 | 22 | 22 | 44 |
|  | 50.00 | 50.00 | 100.00 |
|  | 62.86 | 62.86 | 62.86 |
|  | 31.43 | 31.43 | 62.86 |
| 1 | 13 | 13 | 26 |
|  | 50.00 | 50.00 | 100.00 |
|  | 37.14 | 37.14 | 37.14 |
|  | 18.57 | 18.57 | 37.14 |
| Total | 35 | 35 | 70 |
|  | 50.00 | 50.00 | 100.00 |
|  | 100.00 | 100.00 | 100.00 |
|  | 50.00 | 50.00 | 100.00 |
| Pearson Chi-square = 0.00 Prob =1.0000 | | | |

First row has frequencies; second row has row percentages; third row has column percentages and fourth row has cell percentages

Pearson Chi-square (1) = 0.0000; *p*-value = 1.000

Likelihood-ratio Chi-square (1) = 0.0000; *p*-value = 1.000

Cramér's V = 0.0000

Fisher's exact = 1.000

1-sided Fisher's exact = 0.598

Odds Ratio = (13*22)/(13*22) = 1

**Supplementary Table S3B: Two-way table of Outcome and presence of epidemic clone**

| Epidemic Clone | Mortality | | |
| --- | --- | --- | --- |
|  | 0 | 1 | Total |
| 0 | 19 | 13 | 32 |
|  | 59.38 | 40.62 | 100.00 |
|  | 54.29 | 37.14 | 45.71 |
|  | 27.14 | 18.57 | 45.71 |
| 1 | 16 | 22 | 38 |
|  | 42.11 | 57.89 | 100.00 |
|  | 45.71 | 62.86 | 54.29 |
|  | 22.86 | 31.43 | 54.29 |
| Total | 35 | 35 | 70 |
|  | 50.00 | 50.00 | 100.00 |
|  | 100.00 | 100.00 | 100.00 |
|  | 50.00 | 50.00 | 100.00 |
| Pearson Chi-square = 2.07 Prob = 0.1500 | | | |

First row has frequencies; second row has row percentages; third row has column percentages and fourth row has cell percentages

Pearson chi2(1) = 2.0724; *p*-value = 0.150

Likelihood-ratio chi2(1) = 2.0830; *p*-value = 0.149

Cramér's V = 0.1721

Fisher's exact = 0.230

1-sided Fisher's exact = 0.115

Odds Ratio = (22*19)/(16*13) = 2.009615
